# The Oxford Nanopore MinION: delivery of nanopore sequencing to the genomics community

**DOI:** 10.1186/s13059-016-1103-0

**Published:** 2016-11-25

**Authors:** Miten Jain, Hugh E. Olsen, Benedict Paten, Mark Akeson

**Affiliations:** UC Santa Cruz Genomics Institute and Department of Biomolecular Engineering, University of California, Santa Cruz, CA 95064 USA

## Abstract

Nanopore DNA strand sequencing has emerged as a competitive, portable technology. Reads exceeding 150 kilobases have been achieved, as have in-field detection and analysis of clinical pathogens. We summarize key technical features of the Oxford Nanopore MinION, the dominant platform currently available. We then discuss pioneering applications executed by the genomics community.

## Introduction

Nanopore sequencing was pioneered by David Deamer at the University of California Santa Cruz, and by George Church and Daniel Branton (both at Harvard University). Beginning in the early 1990s, academic laboratories reached a series of milestones towards developing a functional nanopore sequencing platform (reviewed in [1, 2]). These milestones included the translocation of individual nucleic acid strands in single file order [3], processive enzymatic control of DNA at single-nucleotide precision [4], and the achievement of single-nucleotide resolution [5, 6].

Several companies have proposed nanopore-based sequencing strategies. These involve either: the excision of monomers from the DNA strand and their funneling, one-by-one, through a nanopore (NanoTag sequencing (Genia), Bayley Sequencing (Oxford Nanopore)); or strand sequencing wherein intact DNA is ratcheted through the nanopore base-by-base (Oxford Nanopore MinION). To date, only MinION-based strand sequencing has been successfully employed by independent genomics laboratories. Where possible, this review focuses on peer-reviewed research performed using the MinION [1, 7–38].

## DNA strand sequencing using the Oxford Nanopore MinION

Oxford Nanopore Technologies (ONT) licensed core nanopore sequencing patents in 2007, and began a strand sequencing effort in 2010 [2]. At the Advances in Genome Biology and Technology (AGBT) 2012 conference, Clive Brown (Chief Technical Officer of ONT) unveiled the MinION nanopore DNA sequencer, which was subsequently released to early-access users in April 2014 through the MinION Access Program (MAP).

**Fig. 1:**
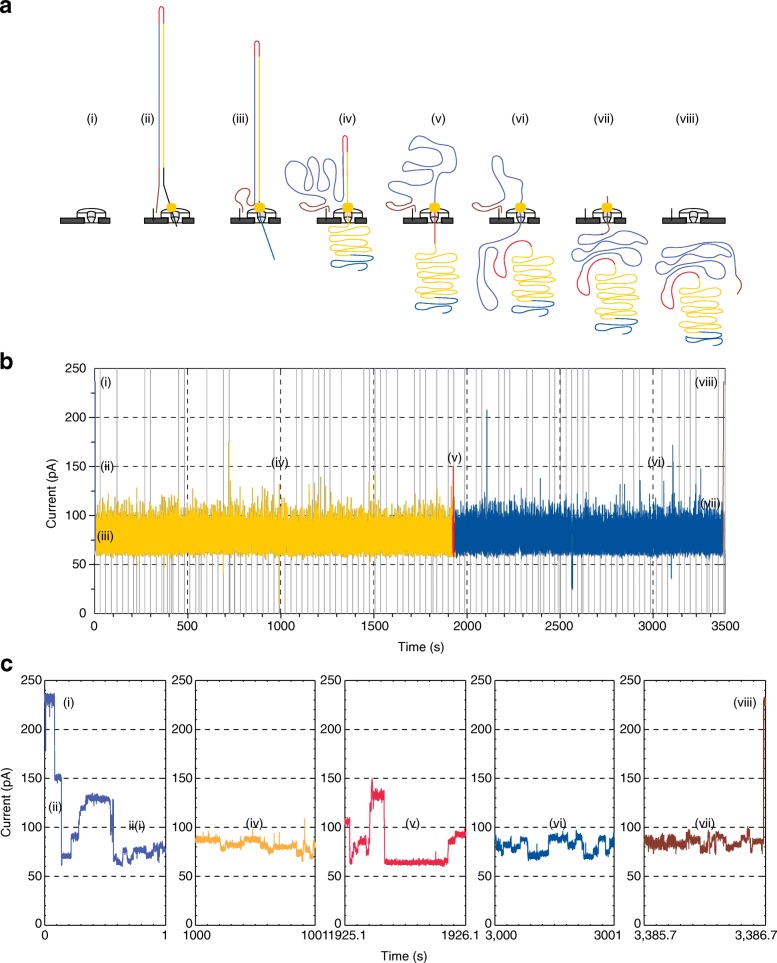
Data for a 2D read of a full-length λ phage dsDNA from the MinION nanopore sequencer. **a** Steps in DNA translocation through the nanopore: (i) open channel; (ii) dsDNA with lead adaptor (*blue*), bound molecular motor (*orange*) and hairpin adaptor (*red*) is captured by the nanopore; capture is followed by translocation of the (iii) lead adaptor, (iv) template strand (*gold*), (v) hairpin adaptor, (vi) complement strand (*dark blue*) and (vii) trailing adaptor (*brown*); and (viii) status returns to open channel. **b** Raw current trace for the passage of the single 48-kb λ dsDNA construct through the nanopore. Regions of the trace corresponding to steps i–viii are labeled. (**c**) Expanded time and current scale for raw current traces corresponding to steps i–viii. Each adaptor generates a unique current signal used to aid base calling

The MinION is a 90-g portable device. At its core is a flow cell bearing up to 2048 individually addressable nanopores that can be controlled in groups of 512 by an application-specific integrated circuit (ASIC). Prior to sequencing, adapters are ligated to both ends of genomic DNA or cDNA fragments (Fig. 1). These adapters facilitate strand capture and loading of a processive enzyme at the 5′-end of one strand. The enzyme is required to ensure unidirectional single-nucleotide displacement along the strand at a millisecond time scale. The adapters also concentrate DNA substrates at the membrane surface proximal to the nanopore, boosting the DNA capture rate by several thousand-fold. In addition, the hairpin adapter permits contiguous sequencing of both strands of a duplex molecule by covalently attaching one strand to the other. Upon capture of a DNA molecule in the nanopore, the enzyme processes along one strand (the ‘template read’). After the enzyme passes through the hairpin, this process repeats for the complementary strand (the ‘complement read’).

As the DNA passes through the pore, the sensor detects changes in ionic current caused by differences in the shifting nucleotide sequences occupying the pore. These ionic current changes are segmented as discrete events that have an associated duration, mean amplitude, and variance. This sequence of events is then interpreted computationally as a sequence of 3–6 nucleotide long kmers (‘words’) using graphical models. The information from template and complement reads is combined to produce a high-quality ‘2D read’, using a pairwise alignment of the event sequences.

An alternate library preparation method does not use the hairpin to connect the strands of a duplex molecule. Rather, the nanopore reads only one strand, which yields template reads. This allows for higher throughput from a flow cell, but the accuracy for these ‘1D reads’ is slightly lower than that of a ‘2D read’.

## Benefits of MinION compared to other next generation sequencing platforms

### Detection of base modifications

Next generation sequencing (NGS) technologies do not directly detect base modifications in native DNA. By contrast, single-molecule sequencing of native DNA and RNA with nanopore technology can detect modifications on individual nucleotides. Previously, Schreiber et al. [39] and Wescoe et al. [40] demonstrated that a single-channel nanopore system can discriminate among all five C-5 variants of cytosine (cytosine (C), 5-methylcytosine (5-mC), 5-hydroxymethylcytosine (5-hmC), 5-formylcytosine (5-fC), and 5-carboxylcytosine (5-caC)) in synthetic DNA. The discrimination accuracies ranged from 92 to 98% for a cytosine of interest in a background of known sequences [40].

In 2016, two research groups independently demonstrated that MinIONs can detect cytosine methylation in genomic DNA [41, 42]. Rand et al. [41] developed a probabilistic method that combines a pair hidden Markov model (HMM) and a hierarchical Dirichlet process (HDP) mixture of normal distributions. They performed a three-way classification among C, 5-mC, and 5-hmC with a median accuracy of 80% in synthetic DNA [41]. Simpson et al. [42] performed a similar study in which they trained an HMM to perform a two-way classification among C and 5-mC, with 82% accuracy in human genomic DNA.

### Real-time targeted sequencing

There are significant advantages to acquiring and analyzing DNA or RNA sequences in a few hours or less, especially for clinical applications. This is difficult using conventional NGS platforms, but relatively straightforward using the MinION because of its size, cost, simple library prep, and portability (see [14]). Beyond this, the MinION platform permits real-time analysis because individual DNA strands are translocated through the nanopore, allowing decisions to be made during the sequencing run.

**Fig. 2:**
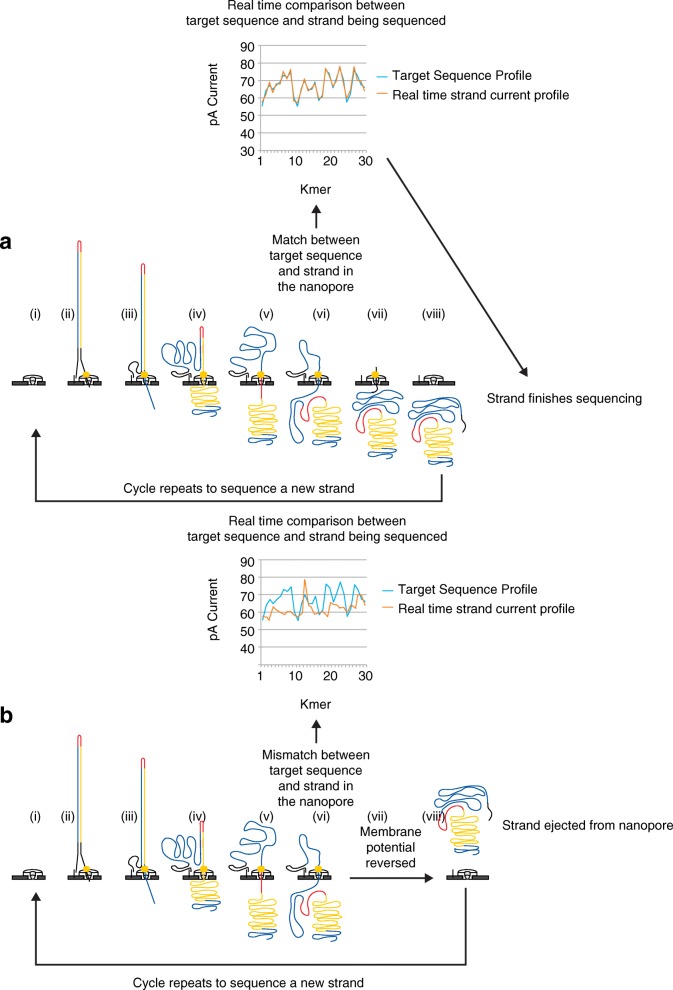
‘Read Until’ strategy for selective sequencing of dsDNA molecules. The ionic current profile obtained during translocation of a DNA strand through the nanopore is compared in real time to the ionic current profile of a target sequence. **a** As sequencing of the template strand of DNA proceeds (during step iv), the measured current is compared to the reference current profile. If there is a match, sequencing of that strand continues to completion (steps v–vii). A new strand can now be captured. **b** Alternatively, if the measured current does not match the reference current profile, the membrane potential is reversed, sequencing of that strand stops, and the strand is ejected (at stage v). A new strand can now be captured. (Image based on the strategy of Loose et al. [43])

This real-time utility of MinION was first demonstrated by Loose et al. [43] in a manuscript that described targeted enrichment (‘Read Until’) of 5 and 10 kb regions from phage lambda double-stranded DNA (dsDNA). Briefly, a mixture of DNA fragments is applied to the MinION flow cell. While a DNA strand is captured and processed in the nanopore, the resulting event levels are aligned against the expected pattern for a target sequence. If the pattern matches, the sequencing continues (Fig. 2a). If the pattern does not match, the DNA strand is ejected from the nanopore so that a subsequent DNA strand can be captured and analyzed (Fig. 2b). In doing this, reads of the targeted strand are rapidly accumulated relative to the DNA strand population as a whole. ‘Read Until’ demonstrates how MinION sequencing could significantly reduce the time required from biological sampling to data inference, which is pertinent for in-field and point-of-care clinical applications.

### Extending read lengths using the MinION

**Fig. 3:**
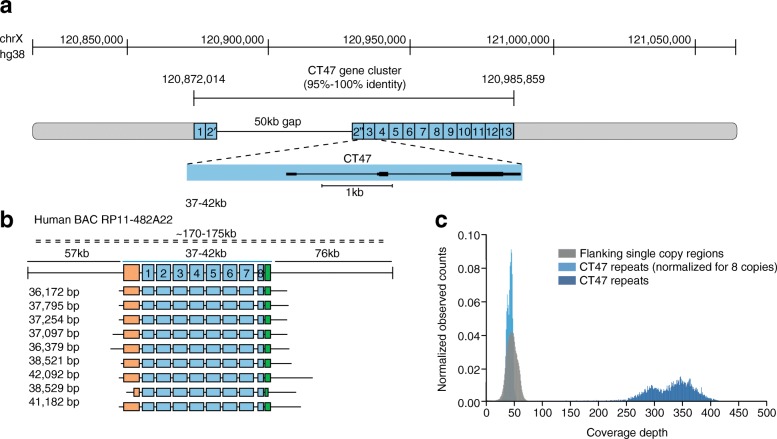
Estimate CT47-repeat copy-number on human chromosome Xq24. **a** BAC end sequence alignments (RP11-482A22: AQ630638 and AZ517599) span a 247-kb region, including 13 annotated CT47 genes [69] (each within a 4.8-kb tandem repeat), and a 50-kb scaffold gap in the GRCh38/hg38 reference assembly. **b** Nine MinION reads from high molecular weight BAC DNA span the length of the CT47-repeat region, providing evidence for eight tandem copies of the repeat. The insert (*dashed line*), whose size is estimated from pulse-field gel electrophoresis, with flanking regions (*black lines*) and repeat region (*blue line*) are shown. Single-copy regions before and after the repeats are shown in *orange* (6.6 kb) and *green* (2.6 kb), respectively, along with repeat copies (*blue*) and read alignment in flanking regions (*gray*). The size of each read is shown to its left. **c** Shearing BAC DNA to increase sequence coverage provided copy-number estimates by read depth. All bases not included in the CT47 repeat unit are labeled as flanking regions (*gray* distribution; mean of 46.2-base coverage). Base coverage across the CT47 repeats was summarized over one copy of the repeat to provide an estimate of the combined number (*dark blue* distribution; mean of 329.3-base coverage) and was similar to single-copy estimates when normalized for eight copies (*light blue* distribution; mean of 41.15-base coverage). (Figure reproduced from Jain et al. [9])

A virtue of nanopore DNA strand sequencing is read lengths that substantially exceed those of dominant NGS platforms. For example, 1D reads over 300 kb in length and 2D reads up to 60 kb in length have been achieved using *Escherichia coli* genomic DNA [44]. To demonstrate utility, Jain et al. [9] used 36-kb + MinION reads to resolve a putative 50-kb gap in the human Xq24 reference sequence. Previously, this gap in the reference sequence could not be completed because it contained a series of 4.8-kb-long tandem repeats of the cancer-testis gene CT47. This work established eight CT47 repeats in this region (Fig. 3).

### Detection of structural variants

Mistakes arising in assemblies of 450-base-long NGS reads are also problematic when characterizing structural variants in human genomes. The problem is acute in cancer, where examples of copy number variants, gene duplications, deletions, insertions, inversions, and translocations are common. For reads that averaged 8 kb in length, Norris et al. [45] used the MinION to detect structural variants in a pancreatic cancer cell line. These authors concluded that the MinION allowed for reliable detection of structural variants with only a few hundred reads compared to the millions of reads typically required when using NGS platforms.

### RNA expression analysis

RNA expression analysis is most often performed by NGS sequencing of cDNA copies. A drawback of this strategy is that the reads are relatively short, thus requiring assembly of cDNA reads into full-length transcripts. This is an issue for the accurate characterization of RNA splice isoforms because there is often insufficient information to deconvolute the different transcripts properly. Full-length cDNA reads would avoid this problem and can be executed with either the PacBio or MinION platforms.

To illustrate, Bolisetty et al. [8] used the MinION to determine RNA splice variants and to detect isoforms for four genes in *Drosophila*. Among these is *Dscam1*, the most complex alternatively spliced gene known in nature, with 18,612 possible isoforms ranging in length from 1806 bp to 1860 bp [8]. They detected over 7000 isoforms for *Dscam1* with >90% alignment identity. Identifying these isoforms would be impossible with 450-base-long NGS reads.

### Bioinformatics and platform advances

The first manuscript to discuss MinION performance was based on limited data and ill-suited analysis, and thus yielded misleading conclusions about the platform’s performance [24]. Over the subsequent 9-month period, ONT optimized MinION sequencing chemistry and base-calling software. Combined with new MinION-specific bioinformatics tools (Table 1), these refinements improved the identity of sequenced reads, that is, the proportion of bases in a sequencing ‘read’ that align to a matching base in a reference sequence, from a reported 66% in June 2014 [9] to 92% in March 2015 [44]. Links to these tools are provided in Table 1 and highlighted in the sections that follow.

**Table 1:** Software tools developed specifically for MinION sequence data; there are existing tools that can also be made to work with nanopore data (not shown)

Name	Applications	Link
Poretools [22]	Sequence data extraction and statistics	https://github.com/arq5x/poretools
poRe [37]	Sequence extraction and basic statistics	https://sourceforge.net/projects/rpore/
BWA MEM [49]	Sequence alignment	https://github.com/lh3/bwa
LAST [48]	Sequence alignment	http://last.cbrc.jp/
NanoOK [20]	Sequence alignment, statistics, and visualization	https://documentation.tgac.ac.uk/display/NANOOK/
marginAlign [9]	Sequence alignment, SNV calling, and statistics	https://github.com/benedictpaten/marginAlign
Nanopolish [50]	Signal alignment and SNV calling	https://github.com/jts/nanopolish
GraphMap [12]	Sequence alignment and SNV calling	https://github.com/isovic/graphmap
minimap	Fast approximate mapping	https://github.com/lh3/minimap
miniasm	De novo assembly	https://github.com/lh3/miniasm
CANU [70]	De novo assembly	https://github.com/marbl/canu
Nanocorrect [48]	De novo assembly	https://github.com/jts/nanocorrect
PoreSeq [53]	De novo assembly and SNV calling	https://github.com/tszalay/poreseq
NaS [23]	De novo assembly	https://github.com/institut-de-genomique/NaS
Nanocorr [13]	De novo assembly	https://github.com/jgurtowski/nanocorr
Mash [71]	Species identification and fast approximate alignments	https://github.com/marbl/mash
minoTour [72]	Real-time data analysis	https://github.com/minoTour/minoTour
Read Until [43]	Selective sequencing	https://github.com/mattloose/RUscripts
Nanocall [46]	Local base-calling	https://github.com/mateidavid/nanocall
DeepNano [47]	Recurrent neural network (RNN)-based base-calling	https://bitbucket.org/vboza/deepnano

*SNV* single nucleotide variant

### De novo base-calling

The base-calling for MinION data is performed using HMM-based methods by Metrichor, a cloud-based computing service provided by ONT. Metrichor presently requires an active internet connection [46, 47] and is a closed source. However, its base-calling source code is now available to registered MinION users under a developer license. To create a fully open-source alternative, earlier in 2016, two groups independently developed base-callers for MinION data. Nanocall [46] is an HMM-based base-caller that performs efficient 1D base-calling locally without requiring an internet connection at accuracies comparable to Metrichor-based 1D base-calling. DeepNano [47], a recurrent neural network framework, performs base-calling and yields better accuracies than HMM-based methods. Being able to perform local, offline base-calling is useful when performing in-field sequencing with limited internet connectivity [30].

### Sequence alignment

**Fig. 4:**
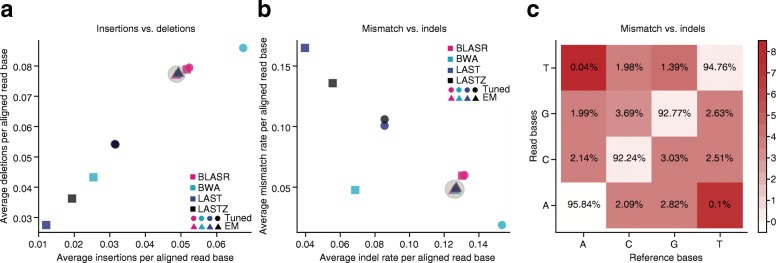
Maximum-likelihood alignment parameters derived using expectation-maximization (*EM*). The process starts with four guide alignments, each generated with a different mapper using tuned parameters. *Squares* denote error estimates derived from different mappers when used without tuning; *circles* denote error estimates post-tuning; and *triangles* denote error estimates post-EM. **a** Insertion versus deletion rates, expressed as events per aligned base. **b** Indel events per aligned base versus rate of mismatch per aligned base. Rates varied strongly between different guide alignments; but EM training and realignment resulted in very similar rates (*gray shading* in *circles*), regardless of the initial guide alignment. **c** The matrix for substitution emissions determined using EM reveals very low rates of A-to-T and T-to-A substitutions. The color scheme is fitted on a log scale, and the substitution values are on an absolute scale. (Figure reproduced from Jain et al. [9])

When the MAP began, the first attempts at aligning MinION reads to reference sequences used conventional alignment programs. Most of these are designed for short-read technologies, such as the 250-nucleotide highly accurate reads produced by the Illumina platform. Not surprisingly, when applied to lower accuracy 10-kb MinION reads, these aligners disagreed in their measurement of read identity and sources of error, despite parameter optimization (Fig. 4). MarginAlign was developed to improve alignments of MinION reads to a reference genome by better estimating the sources of error in MinION reads [9]. This expectation-maximization-based approach considerably improves mapping accuracy, as assayed by improvements in variant calling, and yielded a maximum likelihood estimate of the insertion, deletion, and substitution errors of the reads (Fig. 4). This was later used by a MAP consortium to achieve a 92% read accuracy for the *E. coli* k12 MG1655 genome [44].

MarginAlign refines alignments generated by a mapping program, such as LAST [48] or BWA mem [49], and is therefore reliant on the accuracy of the initial alignment. GraphMap [12] is a read mapper that employs heuristics that are optimized for longer reads and higher error rates. In their study, Sović et al. [12] demonstrated that GraphMap had high sensitivity (comparable to that of BLAST) and that GraphMap’s estimates of error rates were in close agreement with those of marginAlign.

### De novo assembly

The current error profile of MinION reads makes them largely unsuitable for use with de novo assembly methods that are designed for short reads, such as de Bruijn graph-based methods. This is principally for two reasons. First, these methods rely on a sufficient fraction of all possible k-mers sequenced being reconstructed accurately; the overall indel and substitution error rates produced by MinION are unlikely to meet this demand. Second, de Bruijn graphs, in their structure, do not exploit the longer-read information generated by the MinION. Instead, nanopore sequencing is helping to mark a return to overlap-consensus assembly methods [50], a renaissance that largely started with the earlier advent of SMRT sequencing [51]. Overlap-consensus methods were principally developed for lower-error-rate Sanger-based sequencing, and so novel strategies are required to error correct the reads before they are assembled. The first group to demonstrate this approach achieved a single contig assembly of the *E. coli* K-12 MG1655 genome at 99.5% base level accuracy using only MinION data [50]. Their pipeline, ‘nanocorrect’, corrected errors by first aligning reads using the graph-based, greedy partial order aligner method [52], and then by pruning errors that were apparent given the alignment graph. The error-corrected reads were then assembled using the Celera Assembler. This draft assembly was then further improved using Loman and co-worker’s polishing algorithm, ‘nanopolish’ [50].

### Single-nucleotide variant calling

Reference allele bias, the tendency to over-report the presence of the reference allele and under-report non-reference alleles, becomes more acute when the error rate of the reads is higher, because non-reference variants are more likely to be lost in noisy alignments. To overcome this problem for MinION reads, several academic laboratories have developed MinION-specific variant calling tools.

The marginCaller module in marginAlign [9] uses maximum-likelihood parameter estimates and marginalization over multiple possible read alignments to call single nucleotide variants (SNVs). At a substitution rate of 1% (in silico), marginCaller detected SNVs with 97% precision and 97% recall at 60× coverage. Similarly, by optimizing read level alignments, Sović et al. [12] used their GraphMap approach, for accurate mapping at high identity, to detect heterozygous variants from difficult-to-analyze regions of the human genome with over 96% precision. They also used in silico tests to demonstrate that GraphMap could detect structural variants (insertions and deletions of different lengths) with high precision and recall.

Nanopolish [50] uses event-level alignments to a reference for variant calling. This algorithm iteratively modifies the starting reference sequence to create a consensus of the reads by evaluating the likelihood of observing a series of ionic current signals given the reference nucleotide sequence. At each iteration, candidate modifications to the consensus sequence are made and the sequence with the highest likelihood is chosen. At termination of iteration, the alignment of the final consensus to the final reference sequence defines the variants (differences) between the reads and the reference. This approach was used to demonstrate the feasibility of real-time surveillance as part of a study in West Africa in which Quick et al. [30] identified ebola virus sub-lineages using the MinION with ~80% mean accuracy.

PoreSeq [53] is a similar algorithm to Nanopolish, published around the same time, that also iteratively maximizes the likelihood of observing the sequence given a model. Their model, which like Nanopolish uses MinION event-level data, accounts for the uncertainty that can arise during the traversal of DNA through the nanopore. PoreSeq can achieve high precision and recall SNV-calling at low coverages of sequence data. Using a 1% substitution rate in the M13 genome, Szalay and Golovchenko [53] demonstrated that PoreSeq could detect variants with a precision and recall of 99% using 16× coverage. This is around the same accuracy as marginAlign on the same data, but at a substantially lower coverage, demonstrating the power of the event-level, iterative approach.

### Consensus sequencing for high accuracy

The read accuracy of 92% currently achieved by MinION is useful for some applications, but at low coverage it is insufficient for applications such as haplotype phasing and SNV detection in human samples, where the number of variants to be detected is smaller than the published variant-detection error rates of algorithms using MinION data. One method previously used to improve the quality of single-molecule sequence employed rolling circle amplification [51]. In a parallel method for the MinION, Li et al. [54] used rolling circle amplification to generate multiple copies of the 16S ribosomal RNA (rRNA) gene in one contiguous strand. MinION nanopore sequencing of each contiguous strand gave a consensus accuracy of over 97%. This allowed sensitive profiling in a mixture of ten 16S rRNA genes.

## Current applications of the MinION

### Analysis of infectious agents at point-of-care

Next-generation sequencing can detect viruses, bacteria, and parasites present in clinical samples and in a hospital environment [11, 14, 27, 34]. These pathogen sequences enable the identification and surveillance of host adaptation, diagnostic targets, response to vaccines, and pathogen evolution [30]. MinIONs are a new tool in this area that provide substantial advantages in read length, portability, and time to pathogen identification, which is documented to be as little as 6 h from sample collection [14]. Pathogen identification can be performed in as little as 4 min once the sample is loaded on the MinION [14]. The breadth of clinical applications demonstrated to date include studies of chikungunya virus [14], hepatitis virus C [14], *Salmonella enterica* [28], and *Salmonella typhimurium* [7], as well as work on antibiotic resistance genes in five Gram-negative isolates and on the mecA gene in a methicillin-resistant *Staphylococcus aureus* (MRSA) isolate [17].

Arguably, the most inspired clinical use of the MinION to date involved teams of African and European scientists who analyzed ebola samples on-site in West Africa [30, 55]. The recent viral epidemic was responsible for over 28,599 ebola cases and more than 11,299 deaths [56]. In the larger of the two studies, Quick and colleagues [30] transported a MinION field sequencing kit (weighing <50 kg, and fitting within standard suitcases) by commercial airline to West Africa. Once there, they sequenced blood samples from 142 ebola patients in a field laboratory. Ebola virus sequence data were generated within 24 h after sample delivery, with confirmation of ebola sequences taking as little as 15 min of MinION run time. To our knowledge, these studies by Quick et al. [30] and by Hoenen et al. [55] are the first applications of any sequencing device for real-time on-site monitoring of an epidemic.

### Teaching and citizen science

The low cost of entry and portability of the MinION sequencer also make it a useful tool for teaching. It has been used to provide hands-on experience to undergraduate students as part of a recently taught course at Columbia University [57] and to teach graduate students at the University of California Santa Cruz. Every student was able to perform their own MinION sequencing. Similarly, the short and simple process of preparing a sequencing library allowed researchers at Mount Desert Island Biological Laboratory in Maine to train high school students during a summer course and have them run their own MinION experiments. Their Citizen Science initiative intends to address questions pertaining to health and environment that would otherwise be implausible [58].

### Aneuploidy detection

One of the immediate applications of the MinION is aneuploidy detection in prenatal samples. The typical turnaround time for aneuploidy detection in such samples is 1–3 weeks when using NGS platforms [59]. Wei and Williams [38] used the MinION to detect aneuploidy in prenatal and miscarriage samples in under 4 h. They concluded that the MinION can be used for aneuploidy detection in a clinical setting.

### MinIONs in space

At present, it is hard to detect and identify bacteria and viruses on manned space flights. Most of these analyses, along with understanding the effects of space travel on genomes, occur when the samples are brought back to Earth. As a first step to resolve this shortcoming, NASA plans to test MinION-based real-time sequencing and pathogen identification on the International Space Station (ISS) [60, 61]. In a proof-of-concept experiment, Castro-Wallace et al. [62] demonstrated successful sequencing and de novo assembly of a lambda phage genome, an *E. coli* genome, and a mouse mitochondrial genome. They noted that there was no significant difference in the quality of sequence data generated on the ISS and in control experiments that were performed in parallel on Earth [62].

## Outlook

### PromethION

The MinION allows individual laboratories to perform sequencing and subsequent biological analyses, but there is a part of the research community that is interested in high-throughput sequencing and genomics. Realizing this need, ONT has developed a bench-top instrument, PromethION, that is projected to provide high-throughput and is modular in design. Briefly, it will contain 48 flow cells that could be run individually or in parallel. The PromethION flow cells contain 3000 channels each, and are projected to produce up to 6 Tb of sequencing data each day. This equates to about 200 human genomes per day at 30× coverage.

### Read accuracy

Single read accuracy is 92% for the current MinION device [44], which is often sufficient for applications such as the identification of pathogens or mRNA (cDNA) splice variants. However, some medical applications, such as the detection of individual nucleotide substitutions or base adducts in a single mitochondrial genome, would require read accuracies exceeding 99.99%. Given prior experience, it is reasonable that ONT will continue to improve their chemistry and base-calling software. Nevertheless, it is probable that Q40 nanopore sequencing will entail a single strand re-read strategy [2].

As is true for all sequencing platforms, MinION’s base-call accuracy is improved using consensus-based methods. For example, for an *E. coli* strain where single reads averaged ~80% accuracy, consensus accuracy improved to 99.5% at 30× coverage [50]. The remaining 0.5% error appears to be non-random. This improvement is in part due to the inability of the present MinION platform to resolve homopolymers longer than the nanopore reading head (six nucleotides), and to the absence of training in the detection of base modifications. It is plausible that resolving these two issues will push nanopore consensus accuracy to ≥99.99%.

### Read length

With the advent of single-molecule sequencing technologies (PacBio and MinION), the average read lengths increased from 250 nucleotides to 10 kb. More recently, reads of more than 150 kb have routinely been achieved with the MinION (Akeson, unpublished findings), and this is expected to improve in the next few months. Achieving long reads will allow progress in understanding highly complex and repetitive regions in genomes that are otherwise hard to resolve.

### Direct RNA sequencing

Sequencing of direct RNA with nanopore technology is an active area of development at ONT and in academic research groups. Single-molecule detection of tRNA has been previously demonstrated in single-channel and solid-state nanopores [63, 64]. Nanopore sensing can also detect nucleotide modifications in both DNA [39–42] and tRNA [65]. Direct RNA sequencing will reveal insights in RNA biology that presently can get lost due to issues with reverse transcription and PCR amplification.

### Single-molecule protein sensing

At present, mass spectrometry is the preferred technique for performing a comprehensive proteomics analysis [66], but there are limitations to the sensitivity, accuracy, and resolution of any one analytical technique [66]. In 2013, Nivala et al. [67] demonstrated enzyme-mediated translocation of proteins through a single-channel nanopore. Their study showed that sequence-specific features of the proteins could be detected. They then engineered five protein constructs bearing different mutations and rearrangements, and demonstrated that these constructs could be discriminated with accuracies ranging from 86 to 99%. Protein sequencing will allow studies of complex interactions among cells in different tissues [68].

## Conclusions

Nanopore DNA strand sequencing is now an established technology. In the short interval since the ONT MinION was first released, performance has improved rapidly, and the technology now routinely achieves read lengths of 50 kb and more and single-strand read accuracies of better than 92%. Improvement in read lengths, base-call accuracies, base modification detection, and throughput is likely to continue. Owing to its portability, the MinION nanopore sequencer has proven utility at the point-of-care in challenging field environments. Further miniaturization of the platform (SmidgION) and associated library preparation tools (Zumbador, VolTRAX) promise an age of ubiquitous sequencing. Parallel applications, including direct RNA sequencing, are on the horizon.

## Abbreviations

5-hmC: 5-hydroxymethylcytosine
5-mC: 5-methylcytosine
C: Cytosine
dsDNA: Double-stranded DNA
HMM: Hidden Markov model
ISS: International Space Station
MAP: MinION Access Program
NGS: Next generation sequencing
ONT: Oxford Nanopore Technologies
rRNA: Ribosomal RNA
SNV: Single nucleotide variant
