# Erratum to: The Oxford Nanopore MinION: delivery of nanopore sequencing to the genomics community

**DOI:** 10.1186/s13059-016-1122-x

**Published:** 2016-12-13

**Authors:** Miten Jain, Hugh E. Olsen, Benedict Paten, Mark Akeson

**Affiliations:** UC Santa Cruz Genomics Institute and Department of Biomolecular Engineering, University of California, Santa Cruz, CA 95064 USA

## Erratum

After the publication of this work [1] the author noted there was an error in the Outlook section under PromethION where it states:

The MinION allows individual laboratories to perform sequencing and subsequent biological analyses, but there is a part of the research community that is interested in high-throughput sequencing and genomics. Realizing this need, ONT has developed a bench-top instrument, PromethION, that is projected to provide high-throughput and is modular in design. Briefly, it will contain 48 flow cells that could be run individually or in parallel. The PromethION flow cells contain 3000 channels each, and are projected to produce up to 6 Tb of sequencing data each day. This equates to about 200 human genomes per day at 30× coverage.

The last sentence should read: "This equates to over 60 human genomes per day at 30x coverage."

We apologise for this error.
